# Testing the protein-leverage hypothesis using population surveillance data

**DOI:** 10.1098/rsos.220756

**Published:** 2022-09-28

**Authors:** Alistair M. Senior, David Raubenheimer, Stephen J. Simpson

**Affiliations:** ^1^ Charles Perkins Centre, The University of Sydney, Camperdown, NSW 2006, Australia; ^2^ School of Life and Environmental Sciences, The University of Sydney, Camperdown, NSW 2006, Australia; ^3^ School of Mathematics and Statistics, The University of Sydney, Camperdown, NSW 2006, Australia

**Keywords:** appetite, cohort, dietary recall, food frequency, energy, obesity

## Abstract

It is hypothesized that humans exhibit ‘protein leverage’ (PL), whereby regulation of absolute protein intake results in the over-consumption of non-protein food on low percentage protein diets. Testing for PL using dietary surveillance data involves seeking evidence for a negative association between total energy intake and percentage energy from protein. However, it is unclear whether such an association might emerge without PL due to the structure of intake data (protein and non-protein intakes have different means and variances and covary). We derive a set of models that describe the association between the expected estimate of PL and the distributions of protein and non-protein intake. Models were validated via simulation. Patterns consistent with PL will not emerge simply because protein intake has a lower mean and/or variance than non-protein. Rather, evidence of PL is observed where protein has a lower index of dispersion (variance/mean) than non-protein intake. Reciprocally, the stronger PL is the lower the index of dispersion for protein intake becomes. Disentangling causality is ultimately beyond the power of observational data alone. However, we show that one can correct for confounders (e.g. age) in generating signals of PL, and describe independent measures that can anchor inferences around the role of PL.

## 1. Introduction

The way that an organism regulates its intake of nutrients is a core question in metabolic, ecological and evolutionary science. Through the twentieth century, evolutionary ecology was heavily focused on the assumption that the organism is adapted to maximize a single currency, usually energy intake, per unit of time [1]. This focus on energy overlooks the fact that different nutrients have different physiological roles. Working in parallel over this period, medical nutrition science looked beyond energy, to consider how deficits of specific nutrients such as protein and other micronutrients might be affecting health [2]. Medical science, however, paid less attention to the adaptive basis for appetites, and how diseases could be a by-product of the interaction between appetites and the nutritional environment [3].

**Figure 1.**
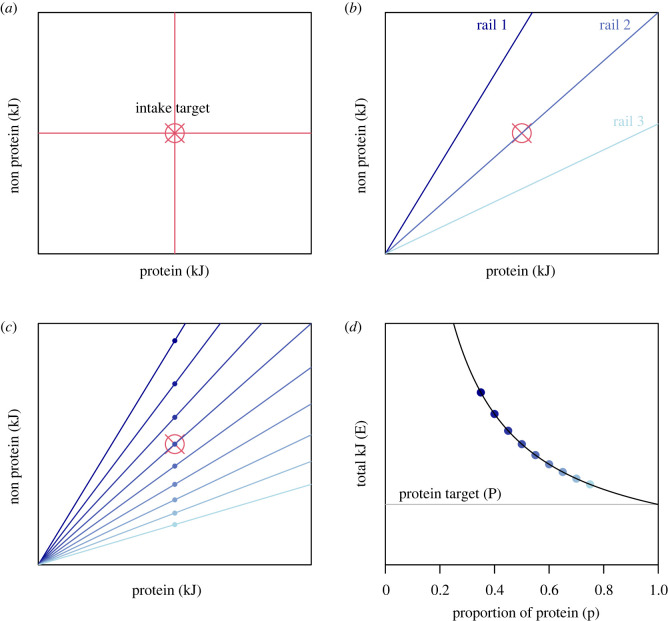
(*a*) A two-dimensional nutrient space denoting intake of protein on the *x*-axis and intake of non-protein (units of energy, kJ) on the *y*-axis. An organism's intake target is shown in red. (*b*) Examples of nutritional rails. The ratio of protein to non-protein in the food/diet increases from rail 1 (dark blue) to rail 3 (light blue). Rail 2 contains a ratio of protein to non-protein identical to the intake target. (*c*) Here the organism strictly defends its intake of protein energy, but as a result consumes an excess of non-protein energy on low protein rails (dark blue) or a deficit on high protein rails (light blue). (*d*) Under strict protein defence, as the proportion of protein in the diet, *p*, increases, total energy intake, *E*, falls exponentially toward the protein target, *P*; the relationship is described by the function *E* = *Pp^L^*, where *L* = −1. This situation is referred to as 'complete protein leverage'.

More recently, the geometric framework for nutrition (GFN) has emerged from evolutionary ecology and is being now used in human nutrition [4]. The GFN combines and builds on the preceding perspectives to understand how appetites have evolved to regulate the intake of multiple nutritional dimensions simultaneously. The GFN adopts a state-space approach, where the intake of *n* nutrients is considered in an *n*-dimensional space (figure 1*a*). A core concept is the intake target. The intake target is a coordinate or region within the space representing the intake of the *n*-nutrients that an organism seeks to achieve by eating the foods available (figure 1*a*). Foods are represented by vectors or ‘nutritional rails’ that project through the space from the origin with slope determined by the ratio of nutrients therein (figure 1*b*). Integrated over a longer timeframe a rail could represent the ratio of nutrients in an organism's diet rather than a single food.

Given ad libitum access to a food/diet with a nutritional ratio that does not match that of the intake target, an organism must compromise between over- and under-consuming different nutrients. One strategy is to strictly regulate the intake of one nutrient at the expense of under/over-consuming others (figure 1*c*). Such a strategy may have important consequences for total energy intake. For example, consider the case where an organism strictly defends its intake of protein energy at the expense of non-protein energy (figure 1*c*). Here, total energy, which is the sum of protein and non-protein intake achieved on a food rail, will fall as the proportion of protein in the rail increases (figure 1*d*); this is the concept of ‘protein leverage’ (PL; [5,6]). The relationship between energy intake and protein content in a food/diet under protein leverage can be described as

1.1 $$E=Pp^{L},$$

where *E* is total energy intake, *p* is the proportion of energy contributed by protein in the diet, *P* is a constant and *L* is the strength of leverage [7,8]. Where the protein target is defended strictly (e.g. figure 1*c*), *L* = −1 and *P* will be equal to the organism's target intake of protein as well as the absolute amount of protein eaten (figure 1*d*). As the protein target becomes less strictly defended, *L* will increase and *P* will deviate from the protein target; *L* = 0 suggests that the protein and non-protein energy are regulated equally strongly and there will be no association between *p* and *E* (i.e. no protein leverage).

Evidence for PL has been observed across numerous animal species in controlled laboratory experiments (see [4,8]). Detailed field studies on non-human primates in which the feeding behaviour of individuals was followed intensively over extended periods have also documented patterns of macronutrient intake consistent with PL (e.g. [9,10]). In addition to such behavioural evidence for PL, laboratory studies have made accelerating progress toward understanding the mechanisms that control protein appetite, both in mammals and invertebrate models (e.g. [11–13]).

The degree to which humans exhibit PL is a critical aspect of our biology with consequences for non-communicable disease. The protein leverage hypothesis (PLH) posits that PL in humans coupled with the dilution of dietary protein in the food supply has contributed to the global obesity epidemic [6,8]. There are challenges in translating inevitably simplified experimental designs conducted under somewhat artificial conditions to the behaviour of free-living humans [14,15]. Nonetheless, randomized controlled trials have demonstrated a causal effect of protein content on food intake in a manner entirely consistent with PL [5,16,17]. While two trials did not find elevated energy intake on a 5% protein diet [18,19], these studies must be interpreted with caution as the levels of protein tested are physiologically unsustainable (see [8]).

Detecting PL in a laboratory setting does not, however, prove that it plays a role in driving energy hyperphagia and obesity in realistic settings. It is therefore important to also test for PL in population data, for example from diet surveys and cohort studies (e.g. [20–22]). These data are typically gathered from surveys, questionnaires, food diaries and related dietary assessment tools. From the pre-existing among-individual variation in the proportion of protein in the diet and total energy intake in these data, one may then make an estimate of the strength of PL. This can be done by directly fitting equation (1.1) and taking the *L* coefficient. Alternatively, in his derivation Hall [7] shows that equation (1.1) can be expressed as

1.2 log⁡(E)=log⁡(P)+L log(p),

which is useful in an applied context as it allows one to test for PL using a standard parametric linear regression of the form

1.3 $$y_{i}=\alpha +\beta x_{i}+\varepsilon_{i},$$

where *y_i_* is the log total intake of the *i*th observation in a dataset, *α* = log(*P*), *β* = *L* (*α* and *β* are coefficients estimated from the data), *x_i_* = log(*p*) for the *i*th observation (i.e. the predictor data), and *ε_i_* is the residual for the *i*th observation. This approach is helpful because linear regression is easily implemented, widely understood and provides a single measure of the strength of leverage (*β* = *L*) that can be compared across studies and with theoretical expectations. Equation (1.3) is particularly useful in an epidemiological context as one can statistically correct for potential confounders (e.g. age or sex) by adding covariates in the form of a multiple regression. Using population data and the approaches suggested above, for example, Saner *et al.* [20] estimate the strength of PL in a cohort of younger people with obesity as *L* = −0.48. While not complete (i.e. *L* > −1) this degree of leverage is strong enough to have played a significant role in the obesity epidemic [7]. We note here that a non-parametric regression could also be implemented to assess the degree to which *x*_i_ and *y_i_* negatively covary (i.e. a signal of PL). However, unlike parametric regression, such models do not provide a succinct and widely comparable single coefficient of the strength of PL, and are not as widely used by epidemiologists.

A question that arises from the estimation of PL using population data is: will a negative association between energy intake and percentage energy from protein arise spuriously from the relative distributions of protein and non-protein energy intake? The answer is not necessarily obvious because: (i) at the population level, protein and non-protein energy tend to be correlated owing to among-individual variation in factors affecting total food intake, (ii) protein and non-protein energy may have different variances and (iii) protein naturally makes up a relatively small proportion of the diet. Here we present a series of theoretical models that examine the expected associations between the strength of PL and population-level statistics on nutrient intake in the absence and presence of true PL. These models are validated using statistical simulation, where we also challenge underlying assumptions. Finally, we discuss how one might holistically assess evidence for/against PL in a population context.

### 1.1. Model 1

All calculations and simulations were performed using R v. 4.1.0 [23], and code is publicly available. Code is maintained at https://github.com/AlistairMcNairSenior/PL_Theory, and an archived version has been released [24].

We begin by deriving an expression for the expected value of *L* as estimated by equation (1.3) as a function of the means and variances of protein and non-protein intake and their covariance. It is critical to note that in this model the explicit assumption that PL is acting is not the starting point. Rather our aim is to see how the value of *L* might be affected by the relative distributions of protein and non-protein intake.

A model of intake that does not begin with the assumption of PL would be one in which protein intake is a random variable *U*, and non-protein intake is a random variable *V* and the two have no explicit causal effect on one another. We can model these intakes as a bivariate normal distribution with means *μ_U_* and *μ_V_*, standard deviations *σ_U_* and *σ_V_* and covariance *σ_UV_* (the correlation between *U* and *V* will be *ρ_UV_* = *σ_UV_*/(*σ_U_σ_V_*)). Positive covariances are expected where there are factors within the population (beyond PL) that generate variance in total food intake (e.g. activity level). We revisit the assumption of normality below.

In this case total energy intake would be the random variable *Z* = *U* + *V*. Because the sum of normal distributions is itself normal, *Z* will be normally distributed with mean and standard deviation

1.4 $$\mu_{Z}=\mu_{U}+\mu_{V},$$

1.5 $$\sigma_{Z}=\sqrt{\sigma_{U}^{2}+\sigma_{V}^{2}+2\sigma_{UV}},$$

and *Z* (total energy intake) and *U* (intake of protein energy) will have covariance, *σ_UZ_*, and correlation, *ρ_UZ_*

1.6 $$\sigma_{UZ}=\sigma_{U(U+V)}=\sigma_{UU}+\sigma_{UV}=\sigma_{U}^{2}+\sigma_{UV},$$

1.7 $$\rho_{UZ}=\frac{\sigma_{UZ}}{\sigma_{U}\sigma_{Z}}.$$

Let the proportion of total energy coming from protein be random variable *W* = *U*/*Z*. *W* is the ratio of two non-central correlated random variables and will therefore, following equation (1.13) in Pham-Gia *et al.* [25], have the probability density function

1.8 $$f_{W}(w)=K\frac{2(1-\rho_{UZ}^{2})\sigma_{U}^{2}\sigma_{Z}^{2}}{\sigma_{Z}^{2}w^{2}-2\sigma_{UZ}+\sigma_{U}^{2}}+f_{F}\left(1,\frac{1}{2},f_{T}(w)\right),$$

where

1.9 $$K=\frac{1}{2\pi \sigma_{U}\sigma_{Z}\sqrt{1-\rho_{UZ}^{2}}}\exp\left[-\frac{\sigma_{Z}^{2}\mu_{U}^{2}-\sigma_{UZ}\mu_{U}\mu_{Z}+\mu_{Z}^{2}\sigma_{U}^{2}}{2(1-\rho_{UZ}^{2})\sigma_{U}^{2}\sigma_{Z}^{2}}\right],$$

1.10 $$f_{T}(w)=\frac{{\left[-\sigma_{Z}^{2}\mu_{U}w+2\sigma_{UZ}(\mu_{Z}w+\mu_{U})-\mu_{Z}\sigma_{U}^{2}\right]}^{2}}{2\sigma_{U}^{2}\sigma_{Z}^{2}(1-\rho_{UZ}^{2})(\sigma_{Z}^{2}w^{2}-2\sigma_{UZ}w+\sigma_{U}^{2})},$$

1.11 $$f_{F}(\theta ,\gamma ,\zeta )=\overset{\infty }{\underset{k=0}{\sum }}\frac{\left(\theta ,k\right)}{\left(\gamma ,k\right)}\cdot \frac{\zeta^{k}}{k!},$$

where equation (1.11) is Kummer's classical confluent hypergeometric function of first kind, which we have estimated computationally using the ‘kummerM’ function in the *fAsianOptions* package in R [26].

The mean, or expected value, of *W* (proportion of energy coming from protein), and its variance will be

1.12 $$\mu_{W}=E(W)=\int wf_{W}(w)\;dw,$$

1.13 $$\sigma_{W}^{2}=\int {\left(w-\mu_{W}\right)}^{2}f_{W}(w)\;dw,$$

and the covariance, and correlation, between *Z* (total energy) and *W*, will be

1.14 $$\sigma_{WZ}=E(WZ)-E(W)E(Z)=E(U)-E(W)E(Z)=\mu_{U}-\mu_{W}\mu_{Z},$$

1.15 $$\rho_{WZ}=\frac{\sigma_{WZ}}{\sigma_{W}\sigma_{Z}}.$$

Here we see that the proportion of energy coming from protein (*W*) and total energy intake (*Z*) will negatively covary (a sign of PL) when the mean of protein intake (*U*) is less than the mean of proportion of energy coming from protein multiplied by the mean of total energy intake.

Estimating the strength of leverage via equation (1.3) requires fitting *W* and *Z* on the log scale. Thus, to get the expected value of *L*, which is the slope from equation (1.3), requires us to find the probability distribution function for the log of *W*. Let the log of *W* be random variable *X*, then the distribution function for *X*, *f_X_*(*x*), using the method of transformation is

1.16 h(w)=log⁡(w)=x,

1.17 $$h^{-1}(x)=e^{x}=w,$$

1.18 $$\frac{dh^{-1}}{dx}=e^{x},$$

1.19 $$f_{X}(x)=f_{W}(h^{-1}(x))\;\frac{dh^{-1}}{dx}=f_{W}(e^{x})e^{x}.$$

The mean and variance of *X* (log proportion energy from protein) can be calculated as

1.20 $$\mu_{X}=E(X)=\int xf_{X}(x)\;dx,$$

1.21 $$\sigma_{X}^{2}=\int {\left(x-\mu_{X}\right)}^{2}f_{X}(x)\;dx.$$

Now treating the log *Z* (total energy intake) as random variable *Y*, a commonly used approximation for the covariance between *X* and *Y* based on the Taylor series expansion is

1.22 $$\sigma_{XY}=\log\left(1+\frac{\sigma_{WZ}}{\mu_{W}\mu_{Z}}\right)=\log\left(1+\frac{\mu_{U}-\mu_{W}\mu_{Z}}{\mu_{W}\mu_{Z}}\right).$$

Finally, based on this covariance the expected slope of equation (1.3), *β*, which is equivalent to *L* in equations (1.1) and (1.2), can be approximated from population means and variances as

1.23 $$E(\beta )=\frac{\sigma_{XY}}{\sigma_{X}^{2}}=\frac{\log(1+((\mu_{U}-\mu_{W}\mu_{Z})/\mu_{W}\mu_{Z}))}{\sigma_{X}^{2}}=E(L).$$

### 1.2. Model 1: numerical results and simulation

**Figure 2.**
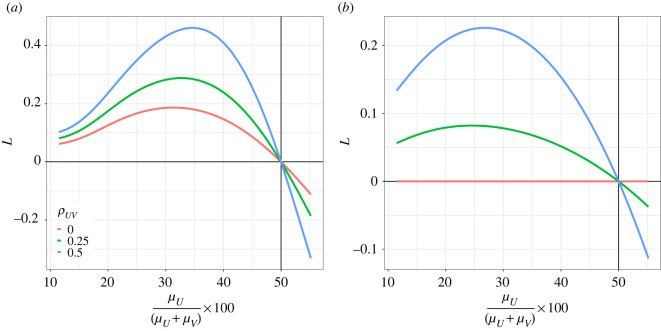
Strength of leverage (*L*) as a function of the mean of protein intake (*μ_U_*) relative to total energy intake (*μ_U_* + *μ_V_*, where *μ_U_* + *μ_V_* has been fixed at 8700) and the correlation between intake of protein and non-protein (*ρ_UV_*), as estimated using equation (1.23). The vertical line indicates where *μ_U_* = *μ_V_* and the horizontal where *L* = 0. In panel (*a*) the variance for both U and V is constant (*σ_U_* = *σ_V_* = 500). In panel (*b*) the index of dispersion (variance normalized to the mean) is held constant (*σ_U_*^2^/*μ_U_* = *σ_V_*^2^/*μ_V_* = 100).

Figure 2*a* shows the expected strength of leverage, *L*, from equation (1.23) as a function of the relative contribution of protein (*μ_U_*) to total energy intake (*μ_U_* + *μ_V_*; assuming *μ_U_* + *μ_V_* = 8700, which is the recommended average adult daily intake in kJ used by Food Standards Australia & New Zealand), and the correlation between protein and non-protein intake (*ρ_UV_*). Here we see that as long as protein contributes less than 50% of total energy on average, we expect to see positive values of *L* in the absence of protein leverage (PL). This analysis indicates that the fact that protein makes up the smaller fraction of total energy, in and of itself, is not enough to drive a spurious correlation that is consistent with the presence of PL (i.e. negative values of *L*).

The analysis in figure 2*a* holds the variance in protein (*U*) and non-protein (*V*) intake constant as the means of each change. However, for many types of data (e.g. lognormally distributed data) there tends to be a positive association between the mean and the variance. Thus, we might expect protein to have lower variance in intake than non-protein if it has a lower mean. We therefore repeated these analyses holding the index of dispersion (ID: variance/mean) for the two nutrients constant. Where there was no correlation between *U* and *V*, *L* was constant and zero regardless of the contribution of protein to total intake (figure 2*b*). Where there is a stronger correlation (and the ID is constant) there are positive values of *L* when *U* tends to make the smaller contribution to energy intake (figure 2*b*). These results suggest that evidence for PL (i.e. *L* < 0) will not arise simply because protein makes up the lower percentage of total energy and has a commensurately lower variance.

**Figure 3.**
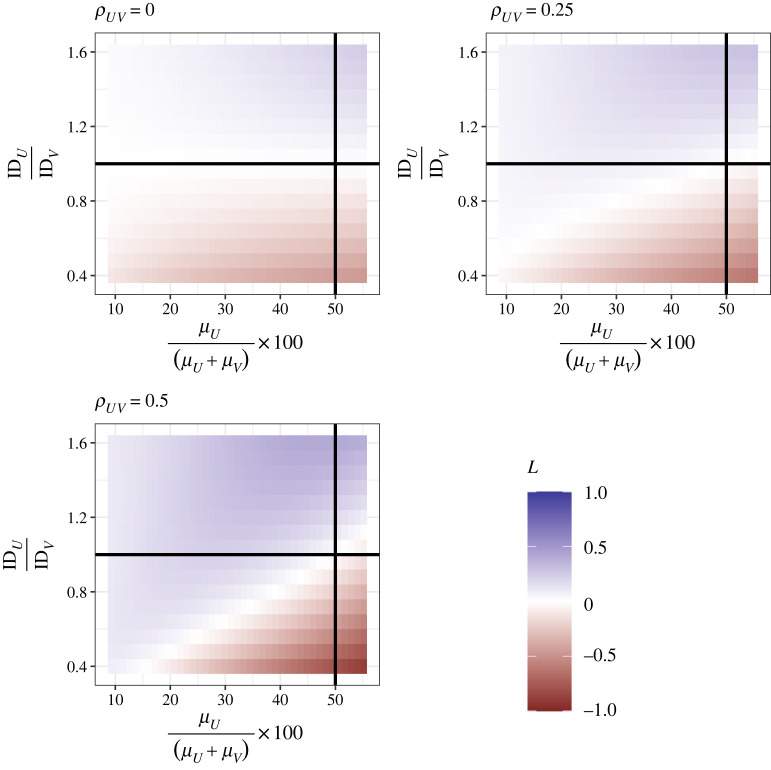
Strength of leverage (*L*) as a function of the mean of protein intake (*μ_U_*) relative to total energy intake (*μ_U_* + *μ_V_*, where *μ_U_* + *μ_V_* has been fixed at 8700), the relative index of dispersion (ID*_U_*/ID*_V_* = [*σ_U_*^2^/*μ_U_*]/[*σ_V_*^2^/*μ_V_*], where ID*_V_* = 125) and the correlation between intake of protein and non-protein (*ρ_UV_*), as estimated using equation (1.23). The horizontal line indicates where ID*_U_* = ID*_V_*, and the vertical line where *μ_U_* = *μ_V_*.

Finally, one might speculate on the value of *L* when protein has a lower mean intake *and* a variance even lower than that expected given the mean (i.e. ID*_U_* < ID*_V_*). Figure 3 shows *L* as a function of the relative contribution of *U* to energy, the relative IDs of *U* and *V*, and the correlation there-between (*ρ_UV_*). Where *ρ_UV_* = 0, apparent indication for PL (*L* < 0) can be expected when the ID for *U* is less than that for *V* (figure 3); put another way, when protein intake has lower than expected variance given its mean, evidence suggesting PL can be observed. As the correlation between protein and non-protein intake increases, negative values of *L* are only estimated when ID*_U_* is substantially smaller than ID*_V_* and/or protein approaches 50% of total energy.

**Figure 4.**
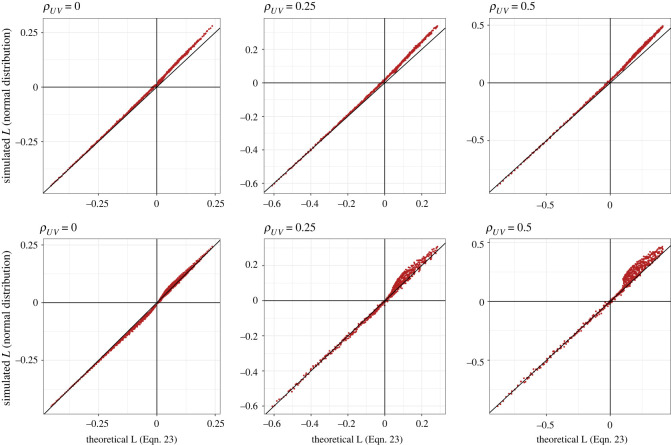
*L* as estimated by equation (1.23) under each condition shown in figure 2 versus *L* as estimated by equation (1.3) fitted to simulated data under those conditions. Top row: data simulated from a bi variate normal distribution. Bottom row: data simulated from a bi variate lognormal distribution.

To test the performance of equation (1.23), we ran a series of statistical simulations. These simulations allow us to evaluate the accuracy of approximations and challenge assumptions, made in the derivation of equation (1.23). For each of the conditions shown in figure 3, we simulated 20 000 protein (*U*) and non-protein (*V*) intakes from a bivariate random-normal distribution using the mvrnorm function in the package *MASS* [27]. From these simulated data we calculated (natural) log total intake (*Y*) and (natural) log proportion of energy from protein (*X*), fitted the model shown in equation (1.3) using the ‘lm’ function in *base* R and extracted the relevant coefficient. The top row of figure 4 shows the value of *L* under each condition as estimated using equation (1.23), and as estimated from simulated data; there is a very strong positive correlation indicating that equation (1.23) performs well. Our approximation and the simulated data match near-perfectly for more negative values of *L*. For more positive values of *L*, equation (1.23) slightly underestimates *L* relative to simulated values (figure 4, top row).

We simulated our intake data from a normal distribution. Because normal distributions are not bound at zero (i.e. can span zero) simulations from them can generate negative values, which become more abundant as the variance increases. This is problematic in the current model because negative values fail in equation (1.3) as log(≤0) is undefined, and consequently any negative simulated values were excluded from our analyses. Furthermore, true intakes of protein and non-protein are ratio scale and cannot be negative (i.e. one cannot eat less than 0 kJ). To test the sensitivity of our results to assumptions of normality we repeated our simulation but using a lognormal distribution (note a lognormal distribution is not the same as the log of a normal distribution; see [28]). Lognormal distributions are useful in the current context because much like real intake data they are bounded at zero (i.e. cannot be negative). Lognormal data were simulated by taking the exponential of data from ‘mvrnorm’ where *ρ_UV_* = 0 and using ‘SimCorRVs’ in the *anySim* package [29] where *ρ_UV_* ≠ 0. There was a tight association between simulated values of *L* and those from equation (1.23). However, where *ρ_UV_* ≠ 0 simulated values were greater than those from equation (1.23) where *L* was positive (figure 4, bottom row). It is important to note that, while equation (1.23) does underestimate *L* > 0, equation (1.23) is near-perfect when *L* is negative, which is the primary area of concern when inferring the presence of PL.

In summary, model 1 indicates that when protein makes up the smaller component of the diet one may detect a pattern consistent with PL only when protein intake has lower-than-expected variance given the mean (figure 3). However, we now demonstrate that such an association need not simply be spurious. Rather, PL is a mechanism by which exactly these distributions are generated.

### 1.3. Model 2

That PL will generate lower variance in protein intake than in non-protein intake can be understood intuitively; PL occurs because protein intake is more strongly regulated than non-protein intake. However, we now present a model that explicitly assumes PL and work up from there to assess how the strength of leverage will affect the relative distributions of protein and non-protein intake.

Taking equation (1.3) as a model of PL, we can begin by assuming that the proportion of energy from protein is a random variable, *W*, that follows a beta distribution, and will then have probability density function

1.24 $$f_{W}(w)=\left[\frac{\Gamma (\kappa +\tau )}{\Gamma (\kappa )\Gamma (\tau )}\right]w^{\kappa -1}{\left(1-w\right)}^{\tau -1},$$

where *κ* and *τ* are the shape parameters. We note that the mean and variance for *W* can be calculated directly from *κ* and *τ* as E(*W*) = *κ*/(*κ* + *τ*) and Var(*W*) = (*κ τ*)/((*κ* + *τ*)^2^+(*κ* + *τ* + 1)). Also, we note that the probability density function for the proportion of energy coming from non-protein, *Q =* 1−*W*, will be simply

1.25 $$f_{Q}(q)=f_{W}(1-q).$$

The log proportion energy from protein, *X* = log(*W*) as is used in equation (1.3) will, following the method of transformation (as in equations (1.16)–(1.19)), have the probability density function *f_X_*(*x*), mean (*μ_X_*) and variance (*σ_X_*^2^)

1.26 $$f_{X}(x)=f_{W}(e^{x})e^{x},$$

1.27 $$\mu_{X}=E(X)=\int xf_{X}(x)\;dx,$$

1.28 $$\sigma_{X}^{2}=\int {\left(x-\mu_{X}\right)}^{2}f_{X}(x)\;dx.$$

Based on equation (1.3) the probability density function for the log of total energy intake, *Y*, will then be the convolution of *f_X_* adjusted for *β* (which is the strength of leverage, *L*) and the probability density function for a random normal distribution *R* with mean = *α* and with (residual) variance *σ_ε_*^2^

1.29 $$f_{R}(r)=\frac{1}{\sigma_{\varepsilon }\sqrt{2\pi }}\exp\left[-\left(\frac{1}{2\sigma_{\varepsilon }^{2}}\right){\left(r-\alpha \right)}^{2}\right],$$

1.30 $$f_{Y}(y)=\int f_{X}(x)\left|\frac{1}{\beta }\right|f_{R}(y-x)\;dx.$$

The mean and variance of *Y* will be

1.31 $$\mu_{Y}=E(Y)=\int yf_{Y}(y)\;dy,$$

1.32 $$\sigma_{Y}^{2}=\int {\left(y-\mu_{Y}\right)}^{2}f_{Y}(y)\;dy.$$

We can then apply the method of transformation to *Y* to get the distribution, mean and variance in energy intake on the natural scale, *Z* as

1.33 $$f_{Z}(z)=f_{Y}(\log z)\frac{d\log z}{dz}=f_{Y}(\log z)\frac{1}{z},$$

1.34 $$\mu_{Z}=\int zf_{Z}(z),$$

1.35 $$\sigma_{Z}^{2}=\int {\left(z-\mu_{Z}\right)}^{2}f_{Z}(z).$$

We can approximate the mean and variance in log absolute protein intake (log(*U*)) as:

1.36 $$\mu_{\log U}=\mu_{X}+\mu_{Y},$$

1.37 $$\sigma_{\log U}^{2}=\sigma_{X}^{2}+\sigma_{Y}^{2}+2\sigma_{XY},$$

1.38 $$\sigma_{XY}=\beta \sigma_{X}^{2}.$$

which back-transforms from the log scale to give expected value (mean) and variance for absolute protein intake, *U*, as

1.39 $$\mu_{U}=\exp\left(\mu_{\log U}+\frac{1}{2}\sigma_{\log U}^{2}\right),$$

1.40 $$\sigma_{U}^{2}=(e^{\sigma_{\log U}^{2}}\;-1)\mu_{U}^{2}.$$

Finally, we can find the mean and variance in non-protein energy intake, *V*. The mean is straightforwardly

1.41 $$\mu_{V}=\mu_{Z}-\mu_{U}.$$

The variance of *V* can be approximated on the log scale (and back-transformed via equation (1.40)) as

1.42 $$\sigma_{\log V}^{2}=\sigma_{S}^{2}+\sigma_{Y}^{2}+2\sigma_{SY},$$

where, *σ_SY_* is the covariance between log total energy intake (log(*Z*) = *Y*) and the log proportion of the energy from non-protein (*S* = log(*Q*) = log(1 − *W*)). The covariance *σ_SY_* can be approximated as

1.43 $$\sigma_{(1-W)Z}=-\sigma_{WZ}=-(E(ZW)-E(Z)E(W))=-(E(U)-E(Z)E(W))=-(\mu_{U}-\mu_{Z}\mu_{W}),$$

1.44 $$\sigma_{SY}=\sigma_{\log(1-W)\log Z}=\log\left(1+\frac{\sigma_{(1-W)Z}}{E(1-W)\mu_{Z}}\right)=\log\left(1+\frac{-(\mu_{U}-\mu_{Z}\mu_{E})}{(1-\mu_{W})\mu_{Z}}\right).$$

The variance in *S* (log proportion of energy from non-protein) can be found as

1.45 $$f_{S}(s)=f_{Q}(e^{s})e^{s},$$

1.46 $$\mu_{S}=E(S)=\int sf_{S}(s)\;ds,$$

1.47 $$\sigma_{S}^{2}=\int {\left(s-\mu_{S}\right)}^{2}f_{S}(s)\;ds.$$

### 1.4. Model 2: numerical results and simulation

**Figure 5.**
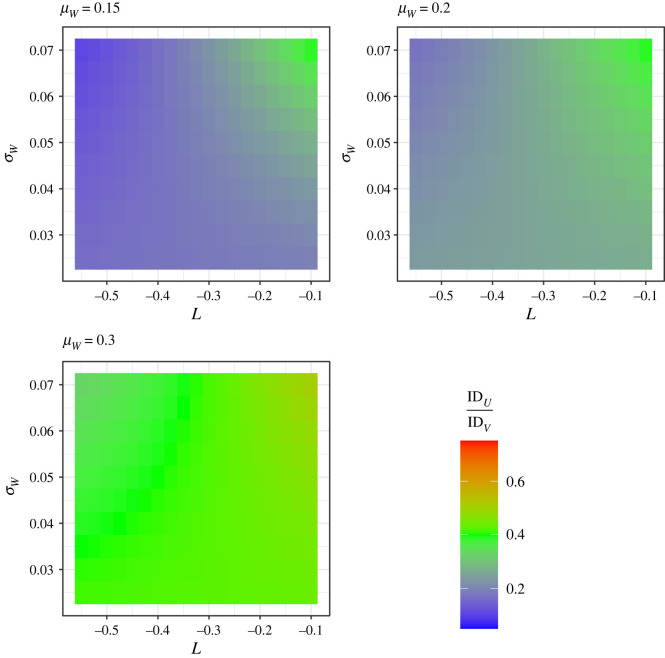
The ratio of the indices of dispersion for protein (*U*) and non-protein (*V*) intake (IDR = ID*_U_*/ID*_V_* = [*σ_U_*^2^/*μ_U_*]/[*σ_V_*^2^/*μ_V_*]) as a function of the strength of leverage (*L* or *β* in equation (1.3)) and the standard deviation in proportion of energy from protein (*σ_W_*). Different values for the mean proportion of energy from protein (*μ_W_*) are assumed across different panels. Model 2 was used to approximate the values of *σ_U_*^2^, *μ_U_*, *σ_V_*^2^ and *μ_V_*. The *α* value for equation (1.3) was fixed at 8700/*μ_W_^L^* in all cases meaning that the modal total intake will be 8700, and residual variance, *σ_ε_*^2^, was fixed at log(8700) * 0.02.

We next calculated the index of dispersion ratio (IDR = ID*_U_*/ID*_V_*) for protein (*U*) and non-protein (*V*) intake as a function of the strength of leverage (*L*), using model 2 to find the relevant means and variances. Figure 5 shows IDR as a function of *L* and the levels of variance in the proportion of energy from protein (*σ_W_*), and assuming protein on average makes up 15%, 20% and 30% of energy. As *L* increases toward 0 (i.e. PL gets weaker) the IDR trends toward 1 (i.e. equal IDs for protein and non-protein). This effect was most marked where we assumed there was higher among-individual variance in proportion of energy from protein (*σ_W_* = 0.07) and protein made up a smaller proportion of total energy (*μ_W_* = 0.15). These results show that stronger leverage will reduce the (mean-corrected) variance in protein intake relative to that for non-protein intake.

**Figure 6.**
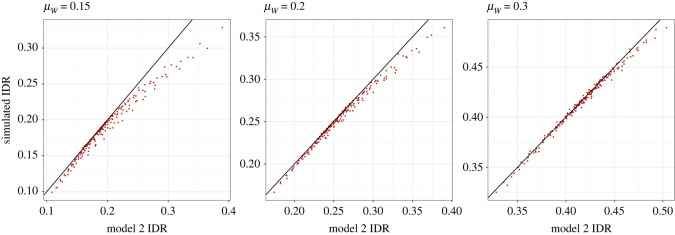
The ratio of the indices of dispersion for protein (*U*) and non-protein (*V*) intake (IDR = ID*_U_* / ID*_V_* = [*σ_U_*^2^/*μ_U_*]/[*σ_V_*^2^/*μ_V_*]) as approximated by model 2, and by simulation, for all parameter values shown in figure 4.

**Figure 7.**
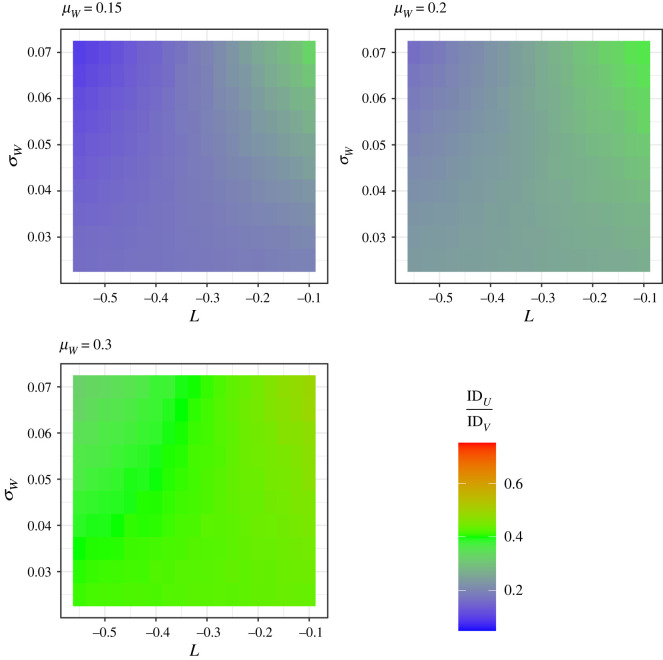
The ratio of the indices of dispersion for protein (*U*) and non-protein (*V*) intake (IDR = ID*_U_*/ID*_V_* = [*σ_U_*^2^/*μ_U_*]/[*σ_V_*^2^/*μ_V_*]) as a function of the strength of leverage (*L*) and the standard deviation in proportion of energy from protein (*σ_W_*). Different values for the mean proportion of energy from protein (*μ_W_*) are assumed across different panels. Simulation was used to estimate the values of *σ_U_*^2^, *μ_U_*, *σ_V_*^2^ and *μ_V_*. The *α* value for equation (1.3) was fixed at 8700/*μ_W_^L^* in all cases meaning that the modal total intake will be 8700, and residual variance, *σ_ε_*^2^, was fixed at log(8700) * 0.02.

We performed a series of simulations to validate model 2. For each set of parameters shown in figure 5 we simulated 100k values of *W* (proportion of energy from protein) from a beta distribution (‘rbeta’ function in *base* R) and 100k residual values from a normal distribution (‘rnorm’ function in *base* R) with mean of 0 and a variance of *σ_ε_*^2^. We then calculated log total energy intake (*Y*) assuming PL via equation (1.3), and absolute total energy intake as *Z* = *e^Y^*. Finally, values of absolute intake of protein and non-protein were calculated as *U* = *ZW* and *V* = *Z*(1−*W*), respectively. The means and variances of *U* and *V* were then used to calculate the simulated IDR. The approximated values of IDR from model 2 and those simulated were strongly correlated (figure 6). However, those from model 2 tended to be slightly higher than the simulated values where *μ_W_* was low and the IDR was also closer to 1 (figure 6). Given this potential discrepancy, we have remade figure 5 using the simulated values of IDR. The trend regarding the effect of *L* on the IDR was the same when calculated via model 2 and via simulation (figure 5 versus figure 7), hence both the analytical model and the simulation yield the same conclusions about the effects of protein leverage on the relative distributions of protein and non-protein intake.

In summary, model 2 shows that PL will lead to a lower index of dispersion (i.e. variance/mean) for protein than non-protein intake.

## 2. Discussion

A negative association between the proportion of dietary energy from protein and total energy intake is expected based on protein leverage (PL). This relationship has been reported in controlled experimental settings, where such an association cannot be explained as a numerical artefact [16]. Our modelling has shown that in analyses of population data such an association will not manifest simply because protein makes up the minor part of total energy intake, nor because protein has a lower variance in intake than non-protein energy (NP). Rather, to produce patterns consistent with PL requires that protein has a variance lower than NP after accounting for the difference in the mean. Human populations typically demonstrate these attributes, with protein making up around 15% of total energy intake, and being less variable around the mean than fat and carbohydrate intake (e.g. [30–32]). As we have shown, PL offers a mechanistic explanation for such patterns; however, the question of causality remains unanswered; do we see what appears to be PL because the variance in protein is lower than that for NP, or vice versa?

It is well established that causality cannot be attributed using population intake data alone (e.g. [33]); hence, the possibility will always remain that some factor other than PL disproportionately reduces the observed variance in protein relative to NP for these types of studies. We can, however, consider what such factors might be.

One possibility is that dietary assessment tools (DAT, e.g. dietary recall, food frequency questionnaires and related online tools) measure protein with higher precision (as distinct from accuracy) than NP, thereby reducing error and variance in protein without the involvement of PL. If this were the case, we would expect the variance in protein to be relatively similar whether measured by objective biomarkers (e.g. urinary nitrogen) or DAT. By contrast, the variance in total energy intake should be reduced when estimated using objective biomarkers such as the doubly labelled water method versus DAT. We tested these predictions using data presented in seven studies which compared biomarker- and DAT-derived values [34–40]. From these studies we have compared the sensitivity of variation in intakes to the method, using the coefficient of variation ratio (CVR: CV_Biomarkers_ / CV_DAT_). The mean CVRs for total energy and protein intake are identical (mean CVR ± s.d., total energy = 0.96 ± 0.09, protein = 0.96 ± 0.13). This, albeit non-exhaustive, survey of the literature suggests that protein intake is measured with a similar degree of precision to total energy intake.

Another possibility is that the difference in variance between NP and protein emerges from heterogeneity in energy requirements among subjects, rather than PL. It is well established that variation in levels of physical activity or changes in basal metabolic rate with age generate variation in energy requirements [41]. Any cross-sectional analysis testing for PL relies on the observed variation in percentage energy from protein representing a ‘natural experiment’; it is assumed that any non-dietary drivers of variance in reported energy intake (e.g. sex, age or socio-economic status or misreporting) are randomly distributed or statistically controlled across the range of percentage protein values within the data. The risk of this kind of confounding is not confined to PL, of course, and epidemiology as a field is well versed in testing for these issues in observational data. In particular, many methods are available for identifying potential under/over-reporting of intakes [42]. The beauty of equation (1.2) as proposed by Hall [7] is that it is readily implemented as a linear regression. This means potential confounders can be corrected using the standard multiple linear regression approaches that are common to all epidemiologists (e.g. adding age or sex, or a misreporting index as a covariate in equation (1.3)), or through stratification and re-analysis (e.g. after removing implausible reporting [42]).

Having corrected for confounders, one approach to give confidence in a causal role for PL is to demonstrate variance in the food environments experienced by individuals within populations. This could be done, for example, by demonstrating variation in the proportions of different categories of foodstuffs eaten. Martínez Steele *et al.* [43] parsed NP and protein from the US National Health and Nutrition Examination Survey (NHANES) data into quintiles for ultra-processed food and beverage consumption (UPF; NOVA Category 4). The NOVA classification system is based on the extent of industrial processing [44] rather than the nutrient compositions of foods and beverages *per se* [45]. This approach is helpful as it provides a classifier one-step removed from the measurement of protein and NP themselves. As predicted by PL, Martínez Steele *et al.* [43] found that protein intakes were consistent across quintiles of UPF intakes, whereas total energy intakes varied greatly. These results in turn accorded with those from a randomized clinical trial [46].

Support for a causal role of PL in affecting total energy intake (as opposed to PL arising spuriously from measurement errors) can also be established if other independently derived objective outcomes known to be affected by total energy intake conform to *a priori* expectations. For example, if lower percentage protein diets truly result in elevated energy intake, higher BMI would be expected on these diets. Other outcomes that might anchor the inference of causality of PL are biomarkers of diet itself, waist–hip ratio, percentage fat mass from DEXA and cardiometabolic markers. Observing that percentage protein shares a negative association with both adiposity and total energy would provide support for the PLH [6,8]. Of course, when tested with population data these more objective outcomes may have their own confounders that must be considered. Three particular issues of note are: (i) the positive association between BMI and under-reporting of non-protein energy intake, which would dampen signal of the PLH [47–49]; (ii) allometries and other complexities of growth and development in younger cohorts [20]; and (iii) if testing for an association with relatively static phenotypes (e.g. BMI or waist-hip ratio) ensuring that the observed nutrient intakes are representative of habitual diet (as opposed to a single measurement period).

Finally, it is worth acknowledging that our models and suggested analyses have been framed around the effects of protein in the diet on energy intake. This is because our work, and that by others, suggests that a two-dimensional model segregating protein and non-protein energy explains a significant amount of variation in food intake and captures features of energy intake overlooked by simpler models [32,50]. However, our insights are readily applicable to any nutritional component that is hypothesized to negatively feedback onto (i.e. leverages) total food and energy intake. This could include specific protein sources (e.g. beef, dairy or plant-derived [19]), certain amino acid combinations and hypothetically even micronutrients (e.g. [51]). Considering the role of specific amino acids in humans will become important as we come to identify the mechanistic bases for protein appetites. It seems increasingly likely that protein appetite is mediated by metabolic signalling that responds to circulating amino acids and the balance of specific amino acids within the dietary protein compartment [11,52,53]. If key amino acid combinations are identified to underpin protein appetites, versions of the analysis targeting these specific nutrients can be implemented. This might involve substituting the proportion of protein in the diet for another proportion entirely (e.g. proportion of non-branched chain amino acids [53]), or fitting the density of specific amino acids within the protein being consumed as a variable that moderates the effect of proportion of protein.

Nowhere is the nature of evidence more disputed than in the field of human nutrition, with diet surveillance data being especially contested [14,15,54–57]. However, to ignore these data altogether risks throwing the proverbial baby out with the bathwater. As discussed, a test of PL using cross-sectional data rests on the assumption that otherwise matched individuals have been subjected to diets differing in the percentage of energy provided as protein; i.e. a ‘natural experiment’. Controlling for factors such as energy requirements and other confounders of percentage dietary protein is the first step toward attempting to meet this assumption. The next step is to associate differences in protein and non-protein energy intakes with data for consumption of key food categories (e.g. UPF, discretionary foods, high-protein foods), thereby seeking a signature for the natural dietary experiment. Finally, predicted associations can be sought for objectively measurable phenotypes known to be causally linked and downstream of excess energy intake (e.g. BMI). These three steps are challenging owing to the usual issues that arise with observational data. However, where these three lines of evidence support a negative estimate for *L* via equation (1.3), one is well-equipped to argue for PL based on population data. Where population data align with pre-clinical/mechanistic literature from animal models, and randomized-controlled trials in people, support for PL converges from across the hierarchy of evidence [8].

## Data Availability

Code is maintained and publicly available from https://github.com/AlistairMcNairSenior/PL_Theory, and an archived version containing the exact analyses within the paper has been publicly released at https://zenodo.org/record/6643746#.Yqka5C8Rpqs (doi:10.528/zenodo.6643746).
